# The happy docs study: a Canadian Association of Internes and Residents well-being survey examining resident physician health and satisfaction within and outside of residency training in Canada

**DOI:** 10.1186/1756-0500-1-105

**Published:** 2008-10-29

**Authors:** Jordan S Cohen, Yvette Leung, Meriah Fahey, Linda Hoyt, Roona Sinha, Lisa Cailler, Kevin Ramchandar, John Martin, Scott Patten

**Affiliations:** 1 University of Calgary Faculty of Medicine, Foothills Medical Center, Assistant Clinical Professor in the Department of Psychiatry, Calgary, Alberta, Canada; 2 University of Calgary, Fellow in the Department of Gastroenterology, Calgary Alberta, Canada; 3 University of Manitoba, Resident in the Department of Obstetrics, Gynecology and Reproductive Sciences, Winnipeg, Manitoba, Canada; 4 Dalhousie University, Resident in the Department of Psychiatry, Halifax, Nova Scotia Canada; 5 University of Alberta, Resident in the Department of Pediatrics, Edmonton, Alberta, Canada; 6 University of British Columbia, Resident in the Department of Radiation Oncology. Vancouver, British Columbia, Canada; 7 McMaster University, Resident in the Department of Radiation Oncology. Hamilton, Ontario, Canada; 8 Memorial University, Resident in the Department of Pediatrics. St. John's, Newfoundland, Canada; 9 University of Calgary Faculty of Medicine, Department of Community Health, Calgary, Alberta, Canada

## Abstract

**Background:**

Few Canadian studies have examined stress in residency and none have included a large sample of resident physicians. Previous studies have also not examined well-being resources nor found significant concerns with perceived stress levels in residency. The goal of "The Happy Docs Study" was to increase knowledge of current stressors affecting the health of residents and to gather information regarding the well-being resources available to them.

**Findings:**

A questionnaire was distributed to all residents attending all medical schools in Canada outside of Quebec through the Canadian Association of Internes and Residents (CAIR) during the 2004–2005 academic years.

In total 1999 resident physicians responded to the survey (35%, N = 5784 residents). One third of residents reported their life as "quite a bit" to "extremely" stressful (33%, N = 656). Time pressure was the most significant factor associated with stress (49%, N = 978). Intimidation and harassment was experienced by more than half of all residents (52%, N = 1050) with training status (30%, N = 599) and gender (18%, N = 364) being the main perceived sources. Eighteen percent of residents (N = 356) reported their mental health as either "fair" or "poor". The top two resources that residents wished to have available were career counseling (39%, N = 777) and financial counseling (37%, N = 741).

**Conclusion:**

Although many Canadian resident physicians have a positive outlook on their well-being, residents experience significant stressors during their training and a significant portion are at risk for emotional and mental health problems. This study can serve as a basis for future research, advocacy and resource application for overall improvements to well-being during residency.

## Findings

The goal of the survey was to increase knowledge of current stressors affecting the well being of residents and to gather information regarding the well-being resources available to them.

## Methods

The CAIR Happy Docs Study was conducted during the 2004–2005 academic year. The CAIR membership list served as the sampling frame for the study. CAIR via its membership represents all resident physicians employed in Canada, with the exception of resident physicians in the province of Quebec. The entire resident population indexed in the sampling frame was considered eligible for inclusion. This included residents from all training programs for all post-graduate years of residency. This voluntary study was distributed to residents in the sampling frame and collected by CAIR board members in each region. Academic days, local mailboxes, e-mail communication, and websites were all tools used to help promote and distribute the surveys [see Additional file 1].

The goals of the study were broad-based and descriptive. The questionnaire was divided into five sections: demographics, stress, intimidation and harassment, well-being and resources. Survey questions included qualitative rating scales, multiple choice responses and yes/no questions. To minimize response acquiescence bias in rating scales responses, the survey included a mixture of positively and negatively stated items [1]. To ensure adequate content validity, the survey stress questions were developed by a study team that consisted of residents and thus contained items that referred to the actual concept of stress that were most germane to residency. The well-being items from the CCHS come from a fully validated instrument developed by Massé and associates [2]. Items derived from Statistics Canada have undergone extensive field-testing and focus group evaluation.

The stress section of the survey contained questions regarding sources of stress as well as methods for dealing with stress. The terms "stress" and "intimidation and harassment" were not formally defined in the survey to allow measurement of "perceived stress" and "perceived intimidation and harassment" which may vary both quantitatively and qualitatively among individuals. Well-being questions were derived from the Canadian Community Health Survey (CCHS) so that results could be compared with members of the general Canadian population [3]. Mental illness screening questions from the CCHS were also included to identify possible psychiatric symptoms in residents. Questions on resources focused on knowledge of current resources, perceived need for future resources, and barriers and limitations to resident physicians seeking aid.

### Statistics

Statistical analysis was conducted with help from the Canadian Post MD Educational Registry (CAPER). Descriptive statistics were used to give an overview of the data as well as for comparison with the CCHS. In cases where not all residents responded to individual questions, percentages fall short of 100%. Percentages reported for different forms of intimidation and harassment reflect the overall percent of residents completing the entire survey and not those that reported intimidation and harassment, except where directly quoted in text. Confidence intervals, Chi-squares, and Fisher exact tests were used to compare differences between groups. P-values less then 0.05 were interpreted as indicating a statistical difference. Where numbers were large enough for valid statistical inference, groups were stratified.

## Results

### (A) Demographics

Overall, 1999 resident physicians responded to the survey, which accounted for 35% of the 5784 residents in the sampling frame. Regional response rates are noted in Table 1 and vary from 28% to 51%. The gender distribution was 47% (N = 942) male and 52% (N = 1043) female. Respondents came from all years of post-graduate training with 31% (N = 612) in their first year, 28% (N = 556) in second year and 42% (N = 831) in their third year of post-graduate medical training or higher.

**Table 1:** Response rate distribution by region

**Region**	**Number and Percent Response**	**Number of Eligible Residents**	**Response Rate **
**Newfoundland**	**N**	196	54
	**%**	100%	28%
			
**Maritimes**	**N**	400	118
	**%**	100%	30%
			
**Ontario**	**N**	3136	1011
	**%**	100%	32%
			
**Manitoba**	**N**	374	127
	**%**	100%	34%
			
**Saskatchewan**	**N**	210	47
	**%**	100%	22%
			
**Alberta**	**N**	813	414
	**%**	100%	51%
			
**British Columbia**	**N**	655	228
	**%**	100%	35%
			
**Total**	**N**	5784	1999
	**%**	100%	35%

Average hours worked per week were 1% (N = 13) less than or equal to thirty five hours per week, 11% (N = 213) worked on average thirty five to fifty hours per week, 26% (N = 514) worked fifty one to sixty five hours per week, 39% (N = 787) worked sixty six to eighty hours per week, and 22% (N = 435) worked more than eighty hours per week.

### (B) Stress

One third of residents reported that most days of there life were "a bit" to "extremely stressful" (33%, N = 656). In the last 12 months, 41% (N = 820) of residents reported most days to be "quite a bit" to "extremely" stressful. Time pressure was the most reported source of stress by residents (Table 2). Overall, males reported less "extreme" stress due to time pressure (33%, N = 310/942) as compared to their female counterparts (41%, N = 432/1043).

**Table 2:** Distribution of factors associated with high stress in residency (scores of 4 or 5 on 5 point scale)

**Source of Stress**	**N**	**Percent (%)**
Time pressure	1403	70
Own work situation	847	43
Financial situation	758	38
Residency program	631	32
Personal relationship	492	24
Own personal or family responsibilities	439	22
Own emotional mental health problem	280	14
Employment status	267	14
Own physical health problem	268	13
Caring for own children	230	12
Caring for others	203	10
Discrimination	128	7
Personal/family safety	91	4

#### Demographic associations with factors contributing to stress

There was a statistically significant relationship between sources of stress and many of the demographic groups of residents (Table 3).

**Table 3:** *Highlighted differences among demographic subgroups contributing to significant resident differences when reporting sources of stress

**Demographic Group**	**Subgroups**	**Possible Stress Factors Contributing to differences in subgroups**
**Age**	< 27 years and 27–30 years	Increased stress reported for all factors except "caring for own children" (e.g. ***time pressure ***69%, N = 662/960)
**Gender**	Men	Increased stress reported for ***finances ***(64%, N = 154/241), ***employment status ***(61%, N = 14/23), and ***discrimination ***(65%, N = 13/20).
	Women	Increased stress for ***mental health ***(55%, N = 23/42)
**Relationship status**	Single	Increased stress with ***personal relationship ***(55%, N = 57/103)
**Location of MD training**	MD outside of Canada	Overall less stress reported, except for ***discrimination ***(65%, N = 13/20)
**Residency year of training**	PGY-3 or above	Increased stress due to ***time pressure ***(42%, N = 413/974), ***financial ***(42%, N = 100/241), ***employment status ***(87%, N = 20/23)
	PGY-1	More stress for ***own work situation ***(48%, N = 102/214)
**Range of hours worked/week**	51–65 & 66–80 average hours per week worked	Increased reporting of ***time pressure ***as a stress (69%, N = 665/961)

* Note: not all potential differences within a demographic group

MD = Medical degree

PGY = postgraduate year

#### Ways of dealing with Stress

Individuals reported both positive and negative coping mechanisms in dealing with stress during their residency training (Table 4). 92% of respondents (N = 1839) spoke with others while 17% (N = 342) used alcohol to cope with stress. Approximately 16% (N = 316) of residents surveyed reported considering changing their residency program. When residents were asked if they could relive their lives, would they pursue another career, 23% (N = 466) said yes.

**Table 4:** Frequency of different responses of dealing with stress (reported by residents as occurring often or sometimes)

**Response to stress**	**N**	**Percent (%)**
Talk to others	1839	92
Relax by doing something enjoyable	1808	90
Look on the bright side of things	1793	89
Jog or do other exercise	1512	76
Wish the situation would go away	1383	70
Avoid being with people	1027	52
Blame yourself	1009	50
Eating more or less than usual	953	47
Sleep more than usual	896	45
Pray or seek spiritual help	758	38
Drinking alcohol	342	17
Using drugs or medication	105	5
Smoking more cigarettes than usual	81	4

### (C) Intimidation and Harassment

**Figure 1:**
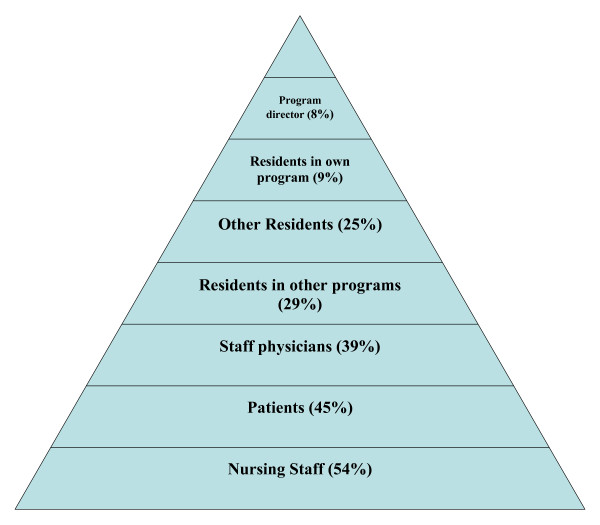
Frequency of groups perceived by residents as being intimidating and harassing to residents.

As seen in Figure 1, residents reported intimidation and harassment most often from nursing staff (54%, N = 1070) and staff physicians (39%, N = 781). The majority of the intimidation and harassment was experienced in the form of inappropriate verbal comments (66%, N = 1330). The perceived basis for the intimidation and harassment was mainly training status (30%, N = 599), or gender (18%, N = 364).

### (D) Well-being

**Figure 2:**
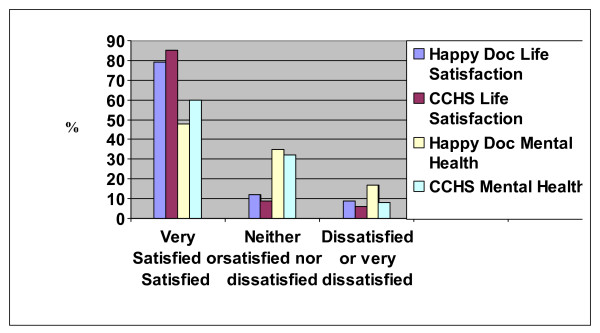
Comparison of relative frequencies of self rated life satisfaction results between residents from the study and the Canadian population.

**Figure 3:**
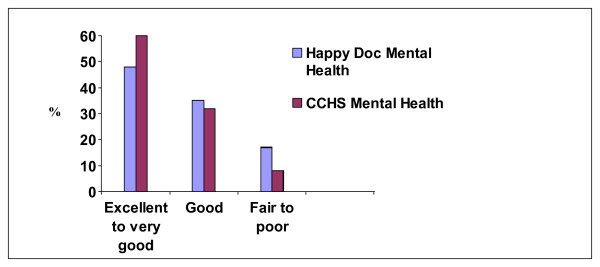
Comparison of relative frequencies of self rated mental health results between residents from the study and the Canadian population.

The results for life satisfaction and self-rated mental health used in this survey are compared to the CCHS estimates (ages 26 to 64 years) for the Canadian population in Figure 2 and 3, respectively.

#### Perceived life satisfaction/well-being

When asked about satisfaction with life in general, 78% (N = 1551) of residents reporting being "satisfied" (Figure 2). The majority of residents reported "excellent" to "very good" (N = 993, 50%), or "good" physical health (N = 662, 33%), with 17% perceiving their physical health as "fair" to "poor" (N = 334). Mental health ratings as compared to the Canadian Community Health Survey are also presented in Figure 3.

#### History of emotional or mental health problems

Almost one-third of residents (30%, N = 607) reported having experienced a mental health problem. Fourteen percent (N = 290) consulted a psychiatrist or psychologist for help in their life. Almost one quarter of residents reported experiencing an emotional or mental health concern during training (23%, N = 453).

#### Psychiatric illness screening

CCHS screening questions were used to identify possible psychiatric symptoms in residents.

Positive predictive values (PPV) of the screening questions for each disorder (generalized anxiety disorder was not completed in CCHS) were used to predict lifetime risk of each psychiatric disorder (Table 5).

**Table 5:** Estimated lifetime risk of psychiatric disorders in residents

**Psychiatric Disorder**	**Number reported; and Percentage**	Positive Predictive Value*	Lifetime Risk* (%)
Depression	1345; 67	0.23	15
Social Phobia	346; 17	0.39	7
Agoraphobia	147; 7	0.85	6
Panic Disorder	622; 31	0.08	2
Mania	238; 12	0.12	1

*Positive predictive values and lifetime risks are based upon positive screens and resultant lifetime risk associated from the Canadian Community Health Survey (CCHS)

#### Significant associations with Mental Health

Residents reporting "poor" to "fair" mental health indicated they deal with stress by drinking alcohol often (6%, N = 21/356), compared to those reporting "good" mental health (2%, N = 11/663), or "excellent" mental health (1%, N = 9/969) [P < 0.001]. Residents reporting "poor" to "fair" mental health indicated they would change residency programs if they could live their life over again, more often (28%, N = 101/356), compared to residents reporting "good" (19%, N = 129/663), or "excellent" mental health (9%, N = 85/969) [P < 0.001]. Residents reporting "poor" to "fair" mental health indicated they would choose a new career (outside of medicine) if they could live life over again (45%, N = 159/356), more often compared to residents reporting good (26%, N = 174/663), and excellent mental health (14%, N = 133/969) [P < 0.001].

Residents reporting "fair" to "poor" mental health were more likely to report financial stress (51%, N = 181/356), compared to residents reporting "good" (43%, N = 284/663), or "excellent" mental health (30%, N = 288/969) [P < 0.001]. Better mental health was associated with being involved in a relationship ("excellent" or "very good" mental health, 58%, N = 562/969; "good", 58%, N = 387/663; compared with "fair" to "poor" mental health, 48%, N = 172/356) [P < 0.001].

### (E) Resources

Residents most frequently reported wanting career counseling (39%, N = 777) and financial counseling (37%, N = 741) as a priority. Program ombudsman and resident support group were also identified as desired resources (27%, N = 545 and 23%, N = 466 respectively). Resident colleague (65%, N = 1305), program director (53%, N = 1066), psychiatrist/psychologists (49%; N = 982), chief resident (45%; N = 904) and support telephone lines (33–44%, N = 667–815) were highest ranked as currently available resources residents would use in a time of personal crisis. When asked what would they do if they suspected that one of their colleagues was experiencing an emotional or mental health problem, most residents reported either suggesting that to their colleague to get help (88%, N = 1754), or offer to escort their colleague to "get help" (75%, N = 1503).

## Conclusion

### Limitations of the Study

Although the survey response rate of 35% was comparable to other Canadian studies like the National Physician Survey and other resident surveys [4,5], it does lead one to question a vulnerability to bias. The results are to some extent more representative of residents who are earlier in their training since junior residents made up half of the sampling frame while 59% of the respondents. Many factors are associated with postgraduate year level that may reflect a response bias leading to an increased reporting of stress (i.e. experience and hours of work). Previous studies have also indicated that the first year of residency is an independent factor contributing to burnout [6]. However, seniority in training has other stresses that may be equally concerning to residents (e.g. final examinations, higher expectations). One must also wonder whether the results are due to a reporting bias among genders, in that females tend to be more open about their stress than their male counterparts. This study did not separate residency specialties and thus we were unable to comment on whether well-being results reflected individual medical residency programs. The stigma of mental illness may have prevented full disclosure and/or abstinence from the survey altogether. Prior studies in medical students have reported poor response rates due to fear of anonymity and confidentiality within the study [7].

Although many Canadian resident physicians have a positive outlook on their well-being, residents experience significant stressors during their training and are at risk for emotional and mental health problems. This study can serve as a basis for future research, advocacy and resource application for overall improvements to well-being during residency.

## Competing interests

All of the authors have in the past or are currently serving as volunteers for the Canadian Association of Internes and Residents.

## Authors' contributions

JC conceived the study, designed the questions and was responsible for coordination of the pilot project in Alberta, which was published in BMC Medical Education in June 2005 [8]. JC, YL, and LH were involved in expanding the pilot project into a national scale survey and coordinated survey distribution through all of the CAIR board members. SP was involved in expanding the pilot project into a national survey, data analysis and editing of the manuscript. All of the authors were involved in the development of the manuscript for publication. YL was involved in the final formatting and editing for submission.

## Supplementary Material

Additional file 1 **The Happy Docs Survey Questions**. Word file with the original questions from the Happy Docs Survey. Click here for file
