# Supplementary material for: The happy docs study: a Canadian Association of Internes and Residents well-being survey examining resident physician health and satisfaction within and outside of residency training in Canada
Source: BMC Res Notes. 2008 Oct 29;1:105. doi: 10.1186/1756-0500-1-105 (PMC2588451; doi:10.1186/1756-0500-1-105)
Supplement: Additional file 1 — The Happy Docs Survey Questions. Word file with the original questions from the Happy Docs Survey. [file 1756-0500-1-105-S1.doc]

**The Happy Doc study**

# The Happy Doc Survey is divided into five sections (A to E). Please complete each section by circling your response or by placing an “X” in the box next to your selected response.

# A. DEMOGRAPHICS

1. (DEM_1) Age: _____________ years

2. (DEM_2) Sex:

| 1 Male | 2 Female |
| --- | --- |

3. (DEM_3) Marital Status:

| 1 Married | 2 Living common-law |
| --- | --- |
| 3 Widowed | 4 Separated |
| 5 Divorced | 6 Single, never married |

4. (DEM_4) Graduate of:

| 1 University In Province Currently Reside In | 2 Canadian University |
| --- | --- |
| 3 Non-Canadian University |  |

5. (DEM_5) Residency Program Category:

1 Technical program (all surgeries including obstetrics and gynecology)

2 Non-technical program (internal medicine, family medicine, psychiatry, pediatrics)

6. (DEM_6) Year of Program:

___1 ___2 ___3 ___4 ___5 ___6 ___7 ___8

7. (DEM_7) On average, how many hours a week do you work in relation to your residency? (include hours on call)

_______ hours

8. (DEM_8) Do you plan to stay in Alberta after completion of your residency program?

1 Yes 2 No 3 Unsure

# B. STRESS

**The following questions deal with stresses that you may experience both personally and in your residency program.**

1. (ST_1) Thinking about the **amount** of stress in your life, would you say that most days are:

| 1 Not at all stressful | 2 Not very stressful |
| --- | --- |
| 3 A bit stressful | 4 Quite a bit stressful |
| 5 Extremely stressful |  |

2. (ST_2) Thinking about the **past 12 months of your residency**, would you say that most days are:

| 1 Not at all stressful | 2 Not very stressful |
| --- | --- |
| 3 A bit stressful | 4 Quite a bit stressful |
| 5 Extremely stressful |  |

3. (ST_3) In general, how would you rate your ability to handle **unexpected and difficult problems**, for example, a family or personal crisis? Would you say your ability is:

1 Excellent 2 Good 3 Fair 4 Poor

4. (ST_4) In general, how would you rate your ability to handle the **day-to-day demands in your life**, for example, handling work, family and volunteer responsibilities? Would you say your ability is:

1 Excellent 2 Good 3 Fair 4 Poor

5. (ST_5) Thinking about stress in your **day to day life**, rate the degree to which you feel each of the following conditions is a source of stress. Please put an **X** in the box that best applies for each statement.

| Possible Sources of Stress in day to day life | **No**  **Stress** |  |  |  |  |  | **Extreme**  **Stress** |
| --- | --- | --- | --- | --- | --- | --- | --- |
|  | **1** | **2** | **3** | **4** | **5** | **6** | **7** |
| Time pressures/not enough time |  |  |  |  |  |  |  |
| Own physical health problem or condition |  |  |  |  |  |  |  |
| Own emotional or mental health problem or condition |  |  |  |  |  |  |  |
| Financial situation (e.g. not enough money, debt) |  |  |  |  |  |  |  |
| Own work situation (e.g. hours of work, working conditions) |  |  |  |  |  |  |  |
| Residency Program |  |  |  |  |  |  |  |
| Employment status |  |  |  |  |  |  |  |
| Caring for-own children (if you have children) |  |  |  |  |  |  |  |
| Caring for-others |  |  |  |  |  |  |  |
| Other personal or family responsibilities |  |  |  |  |  |  |  |
| Personal relationships |  |  |  |  |  |  |  |
| Discrimination |  |  |  |  |  |  |  |
| Personal and family’s safety |  |  |  |  |  |  |  |
| Other (specify) |  |  |  |  |  |  |  |

6. (ST_6) What would you say is the most important thing contributing to feelings of stress you may have? **(Select only one)**

| 1 Time pressures/not enough time | 2 Own physical health problem or condition |
| --- | --- |
| 3 Own emotional or mental health problem or condition | 4 Financial situation (e.g. not enough money, debt) |
| 5 Own work situation (hours of work, working conditions) | 7 School (i.e. residency program) |
| 8 Employment status | 9 Caring for-own children |
| 10 Caring for-others | 11 Other personal or family responsibilities |
| 12 Personal relationships | 13 Discrimination |
| 14 Personal and family’s safety | 15 Other – specify |

7. (ST_7) The following statements may describe stresses that **relate directly to your residency** during the past 12 months. Please indicate whether you **strongly agree, agree, neither agree nor disagree, disagree, or strongly disagree**.

| **Statement** | **Strongly agree** | **Agree** | **Neither agree nor disagree** | **Disagree** | **Strongly disagree** |
| --- | --- | --- | --- | --- | --- |
| Your residency required that you learn new things |  |  |  |  |  |
| Your residency required a high level of skill |  |  |  |  |  |
| Your residency allowed you freedom to decide how you did your job |  |  |  |  |  |
| Your residency required that you do things over and over |  |  |  |  |  |
| Your residency was very hectic |  |  |  |  |  |
| You were free from conflicting demands that others made |  |  |  |  |  |
| You had a lot to say about what happened in your residency |  |  |  |  |  |
| You were exposed to hostility or conflict from the people you worked with |  |  |  |  |  |
| Your supervisor was helpful |  |  |  |  |  |
| There was pressure to conduct research/publish |  |  |  |  |  |
| There was pressure of examinations and evaluations (volume, complexity & time pressure) |  |  |  |  |  |
| There was insufficient sleep & frequent call |  |  |  |  |  |
| Pressure from clinical workload (“scut” work, patient load, too much medical records work) |  |  |  |  |  |
| Stress of high rates of death among patients |  |  |  |  |  |

8. (ST_8) People have different ways of dealing with stress. Thinking about the **ways you deal with stress**, how often do you do each of the following:

|  | **Often** | **Sometimes** | **Rarely** | **Never** |  |
| --- | --- | --- | --- | --- | --- |
| Talk to others |  |  |  |  |  |
| Avoid being with people |  |  |  |  |  |
| Sleep more than usual |  |  |  |  |  |
| Try to feel better by eating more or less than usual |  |  |  |  |  |
| Try to feel better by smoking more cigarettes than usual |  |  |  |  | Do not smoke |
| Try to feel better by drinking alcohol |  |  |  |  |  |
| Try to feel better by using drugs or medication |  |  |  |  |  |
| Try to look on the bright side of things |  |  |  |  |  |
| Jog or do other exercise |  |  |  |  |  |
| Pray or seek spiritual help |  |  |  |  |  |
| Relax by doing something enjoyable |  |  |  |  |  |
| Try to look on the bright side of things |  |  |  |  |  |
| Blame yourself |  |  |  |  |  |
| Wish the situation would go away or somehow be finished |  |  |  |  |  |

9. (ST_9) If possible, I would consider changing my residency program

| 1 Yes | 2 No |
| --- | --- |

10. (ST_10) If I had it to do all over again, I would pursue another career

| 1 Yes | 2 No |
| --- | --- |

## C. INTIMIDATION AND HARRASSMENT

**The following questions relate to intimidation or harassment that you may have experienced during your residency. Keep in mind that all survey information is confidential.**

| 1. (IH1) During your residency, have you ever experienced intimidation or harassment from: | Yes | No |
| --- | --- | --- |
| Program director |  |  |
| Staff physician |  |  |
| Other residents |  |  |
| Nursing staff |  |  |
| Residents in your program |  |  |
| Residents from other programs |  |  |
| Patients |  |  |
| Other (specify)_____________________________________________ |  |  |
| If you have answered **“No”** to **all of IH1, Go to IH6** | | |

2. (IH2) In what **form** did you experience the intimidation or harassment?

| 1 Inappropriate verbal comments | 2 Inappropriate or unwanted physical contact |
| --- | --- |
| 3 Sexual harassment | 4 Work as punishment |
| 5 Privileges/opportunities taken away | 6 Recrimination for reporting |
| 7 Other (specify)__________________________________________________________ | |

3. (IH3) What do **you believe** was the basis for the intimidation or harassment?

| 1 Gender | 2 Language |
| --- | --- |
| 3 Culture | 4 Ethnicity |
| 5 Sexual orientation | 6 Other (specify)_______________________________ |

4. (IH5) How **often** did the intimidation or harassment occur?

| 1 Once | 2 More than once |
| --- | --- |

5. (IH6) Are you aware of the **process** to address issues of intimidation and harassment in your residency?

| 1 Yes | 2 No (**go to Section D**) |
| --- | --- |

6. (IH7) Do you feel that the process is **adequate, fair and independent**?

| 1 Yes | 2 No |
| --- | --- |

#### D. WELL-BEING

**The next questions are about your well-being and areas of your life that could affect your physical and emotional health. Take your time to think about each question before answering.**

1. (MH1) How **satisfied** are you with your life in general?

| 1 Very satisfied | 2 Satisfied |
| --- | --- |
| 3 Neither satisfied nor dissatisfied | 4 Dissatisfied |
| 5 Very dissatisfied |  |

2. (MH2) In general, would you say your **physical** health is:

| 1 Excellent | 2 Very good |
| --- | --- |
| 3 Good | 4 Fair |
| 5 Poor |  |

3. (MH3) In general, would you say your **mental** health is:

| 1 Excellent | 2 Very good |
| --- | --- |
| 3 Good | 4 Fair |
| 5 Poor |  |

4. (MH4) Do you have a family physician?

| 1 Yes | 2 No |
| --- | --- |

5. (MH5) Have you had an appointment with your family physician in the past 12 months?

| 1 Yes | 2 No |
| --- | --- |

6. (MH6) Have you **ever** had emotional or mental health problems?

| 1 Yes | 2 No |
| --- | --- |

7. (MH7) **If yes**, have you sought or received help for this?

| 1 had emotional or mental health problems of no importance | 2 have not sought help, though I have been in need of this |
| --- | --- |
| 3 have consulted GP | 4 have consulted psychologist/psychiatrist |
| 5 have been admitted to hospital | 6 have attended self-help group (e.g. AA) |
| 7 have received complementary or alternative therapy |  |

8. (MH8) Have you had emotional or mental health problems **during residency**?

| 1 Yes | 2 No |
| --- | --- |

9. (MH9) **If yes**, have you sought or received help for this?

| 1 had emotional or mental health problems of no importance | 2 have not sought help, though I have been in need of this |
| --- | --- |
| 3 have consulted GP | 4 have consulted psychologist/psychiatrist |
| 5 have been admitted to hospital | 6 have attended self-help group (e.g. AA) |
| 7 have received complementary or alternative therapy |  |

10. (SCR_1) During your life, have you **ever** had an **attack of fear or panic** when all of a sudden you felt very frightened, anxious or uneasy?

1 Yes (Go to SCR_3) 2 No 3 Don’t know

11. (SCR_2) Have you **ever** had an attack when **all of a sudden**, you became very uncomfortable, you either became short of breath, dizzy, nauseous or your heart pounded, or you thought that you might lose control, die or go crazy?

1 Yes 2 No Don’t know

12. (SCR_3) Have you **ever** in your life had a period **lasting several days or longer** when most of the day you felt **sad, empty or depressed**?

1 Yes 2 No (Go to SCR_5) 3 Don’t know (Go to SCR_5)

13. (SCR_4) Have you **ever** had a period lasting **2 years or longer** when **most days** you felt either **sad or depressed** about how things were going in your life?

1 Yes (Go to SCR_8) 2 No 3 Don’t know

14. (SCR_5) Have you **ever** had a period **lasting several days or longer** when most of the day you were very **discouraged** about how things were going in your life?

1 Yes 2 No (Go to SCR_7) 3 Don’t know (Go to SCR_7)

15. (SCR_6) Have you **ever** had a period lasting **2 years or longer** when **most days** you felt either very **discouraged** about how things were going in your life?

1 Yes (Go to SCR_8) 2 No 3 Don’t know

16. (SCR_7) Have you **ever** had a period lasting **several days or longer** when you **lost interest** in most things you usually enjoy like work, hobbies and personal relationships?

1 Yes 2 No 3 Don’t know

17. (SCR_ 8) Some people have periods lasting **several days or longer** when they feel much more excited and full of energy than usual. Their minds go too fast. They talk a lot. They are very restless or unable to sit still and they sometimes do things that are unusual for them. For example, they may drive too fast or spend too much money.

(SCR_9) During your life, have you **ever** had a period like this lasting **several days or longer**?

1 Yes 2 No 3 Don’t know

18. (SCR_10) Have you **ever** had a period lasting **several days or longer** when most of the time you were very **irritable, grumpy or in a bad mood**?

1 Yes 2 No (Go to SCR_12) 3 Don’t know

19. (SCR_11) Have you **ever** had a period lasting **several days or longer** when most of the time you were so irritable that you either started arguments, shouted at people or hit people?

1 Yes 2 No 3 Don’t know

20. (SCR_12) Did you **ever** have a time in your life when you were a **“worrier”**; that is, when you worried a lot more about things than other people with the same problems as you?

1 Yes (Go to SCR_14) 2 No 3 Don’t know

21. (SCR_13) Did you **ever** have a time in your life when you were much more **nervous or anxious** than most other people with the same problems as you?

1 Yes 2 No Don’t know

22. (SCR_14) Did you **ever** have a period lasting **6 months or longer** when you were anxious and worried **most days**?

1 Yes 2 No 3 Don’t know

23. (SCR_15) Was there **ever** a time in your life when you felt very afraid or **really, really** shy with people, for example meeting new people, going to parties, going on a date or using a public bathroom?

1 Yes (Go to SCR_17) 2 No 3 Don’t know

24. (SCR_16) Was there **ever** a time in your life when you felt very afraid or uncomfortable when you had to do something in front of a group of people, like giving a speech or speaking in class?

1 Yes 2 No (Go to SCR_20) 3 Don’t know (Go to SCR_20)

25. (SCR_17) Was there **ever** a time in your life when you became **very upset or nervous** whenever you were in social situations/when you had to do something in front of a group?

1 Yes 2 No 3 Don’t know

26. (SCR_18) Because of your fear, did you **ever** stay away from social situations/situations where you had to do something in front of a group whenever you could?

1 Yes 2 No 3 Don’t know

27. (SCR_19) Do you think your fear was **ever** much stronger than it should have been?

1 Yes 2 No 3 Don’t know

28. (SCR_20) Was there **ever** a time in your life when you felt very uncomfortable or afraid of either being in **crowds**, going to **public places**, traveling **by yourself**, or traveling **far away from home**?

1 Yes 2 No (Go to Section E) 3 Don’t know (Go to Section E)

29. (SCR_21) Was there **ever** a time in your life when you became very upset or nervous whenever you were in crowds, public places, or traveling?

1 Yes 2 No 3 Don’t know

30. (SCR_22) Because of your fear, did you **ever** stay away from these situations whenever you could?

1 Yes 2 No 3 Don’t know

31. (SCR_23) Do you think your fear was **ever** much stronger than it should have been?

1 Yes 2 No 3 Don’t know

**E. RESOURCES**

1. (RE1) The following resources are available to residents. Please indicate which ones you aware of:

| 1 Phone in hotline (anonymous & confidential for residents) | 2 Physician and Family Support Program |
| --- | --- |
| 3 Resident advocates In Provincial Housestaff Organizations | 4 External psychiatrists or psychologists |
| 5 Emergency consultation/assessment system | 6 Program Director |
| 7 Chief Resident | 8 Resident Colleague |
| 9 Calgary Health Region | 10 University of Calgary |
| 10 Other (specify)__________________________________________________________ | |

2. (RE2) Are there **other programs or resources** that you would like to see available to residents and/or their families? **Mark all that apply.**

| 1 National Help Line | 2 Out of province connection to mental health team |
| --- | --- |
| 3 Spiritual counselor | 4 Program ombudsman (i.e. representative not directly involved in your program to be an advocate) |
| 5 Resident support group (run by residents or possibly psychiatry staff) | 6 Family support program |
| 7 Financial counseling | 8 Career counselling |
| 9 Other ( please specify) _____________________________________________________________ | |

3. (RE3) How important are **each** of the following resources for dealing with emotional or mental health problems?

|  | **Not**  **Important** |  |  |  |  |  | **Extremely**  **Important** |
| --- | --- | --- | --- | --- | --- | --- | --- |
|  | **1** | **2** | **3** | **4** | **5** | **6** | **7** |
| Phone in hotline for residents |  |  |  |  |  |  |  |
| Physician and Family Support Hotline |  |  |  |  |  |  |  |
| External psychiatrists or psychologists |  |  |  |  |  |  |  |
| Emergency consultation/assessment system |  |  |  |  |  |  |  |
| Program Director |  |  |  |  |  |  |  |
| Chief Resident |  |  |  |  |  |  |  |
| Resident Colleague |  |  |  |  |  |  |  |
| Other (specify |  |  |  |  |  |  |  |

4. (RE4) If you were in a situation where you were experiencing an emotional or mental health problem, how would you deal with it? **Please rank your top 3 choices (1,2,3)** **only.**

____ Phone in hotline (anonymous and confidential for residents)

____ Physician and Family Support Hotlines

____ Resident advocates from Provincial Housestaff Organization

____ External psychiatrists or psychologists

____ Emergency consultation/assessment system

____ Program Director

____ Chief Resident

____ Resident Colleague

____ Do nothing

____ Other (specify)____________________________________________________________________

5. (RE5) If you would not seek help for an emotional or mental health concern, **why?**

6. (RE6) If you were aware of a fellow resident who was experiencing an emotional health problem, how would you respond? **Mark all that apply**

1 Suggest resident get help

2 Offer to go with the resident for help

3 Contact program director

4 Notify PGME office

5 Notify Professional Housestaff Organzation for support

6 Notify Royal College of Physicians and Surgeons

7 Contact Provincial Medical Association

8 Do nothing

9 Other (specify)_______________________________________________________________________________

Do you have any comments about your experiences during residency that were not addressed in this survey?

**Thank-you for your participation in this survey.**
